# Polarization spectroscopy methods in the determination of interactions of small molecules with nucleic acids – tutorial

**DOI:** 10.3762/bjoc.14.5

**Published:** 2018-01-08

**Authors:** Tamara Šmidlehner, Ivo Piantanida, Gennaro Pescitelli

**Affiliations:** 1 Division of Organic Chemistry and Biochemistry, Ruđer Bošković Institute; P. O. Box 180, 10002 Zagreb, Croatia; 2 Department of Chemistry, University of Pisa, via Moruzzi 13, Pisa, Italy

**Keywords:** circular dichroism, emission-based dichroism, groove binding, intercalation, linear dichroism, non-covalent interactions, nucleic acids recognition, vibrational circular dichroism

## Abstract

The structural characterization of non-covalent complexes between nucleic acids and small molecules (ligands) is of a paramount significance to bioorganic research. Highly informative methods about nucleic acid/ligand complexes such as single crystal X-ray diffraction or NMR spectroscopy cannot be performed under biologically compatible conditions and are extensively time consuming. Therefore, in search for faster methods which can be applied to conditions that are at least similar to the naturally occurring ones, a set of polarization spectroscopy methods has shown highly promising results. Electronic circular dichroism (ECD) is the most commonly used method for the characterization of the helical structure of DNA and RNA and their complexes with ligands. Less common but complementary to ECD, is flow-oriented linear dichroism (LD). Other methods such as vibrational CD (VCD) and emission-based methods (FDCD, CPL), can also be used for suitable samples. Despite the popularity of polarization spectroscopy in biophysics, aside several highly focused reviews on the application of these methods to DNA/RNA research, there is no systematic tutorial covering all mentioned methods as a tool for the characterization of adducts between nucleic acids and small ligands. This tutorial aims to help researchers entering the research field to organize experiments accurately and to interpret the obtained data reliably.

## Review

### 1. Introduction

**Figure 1:**
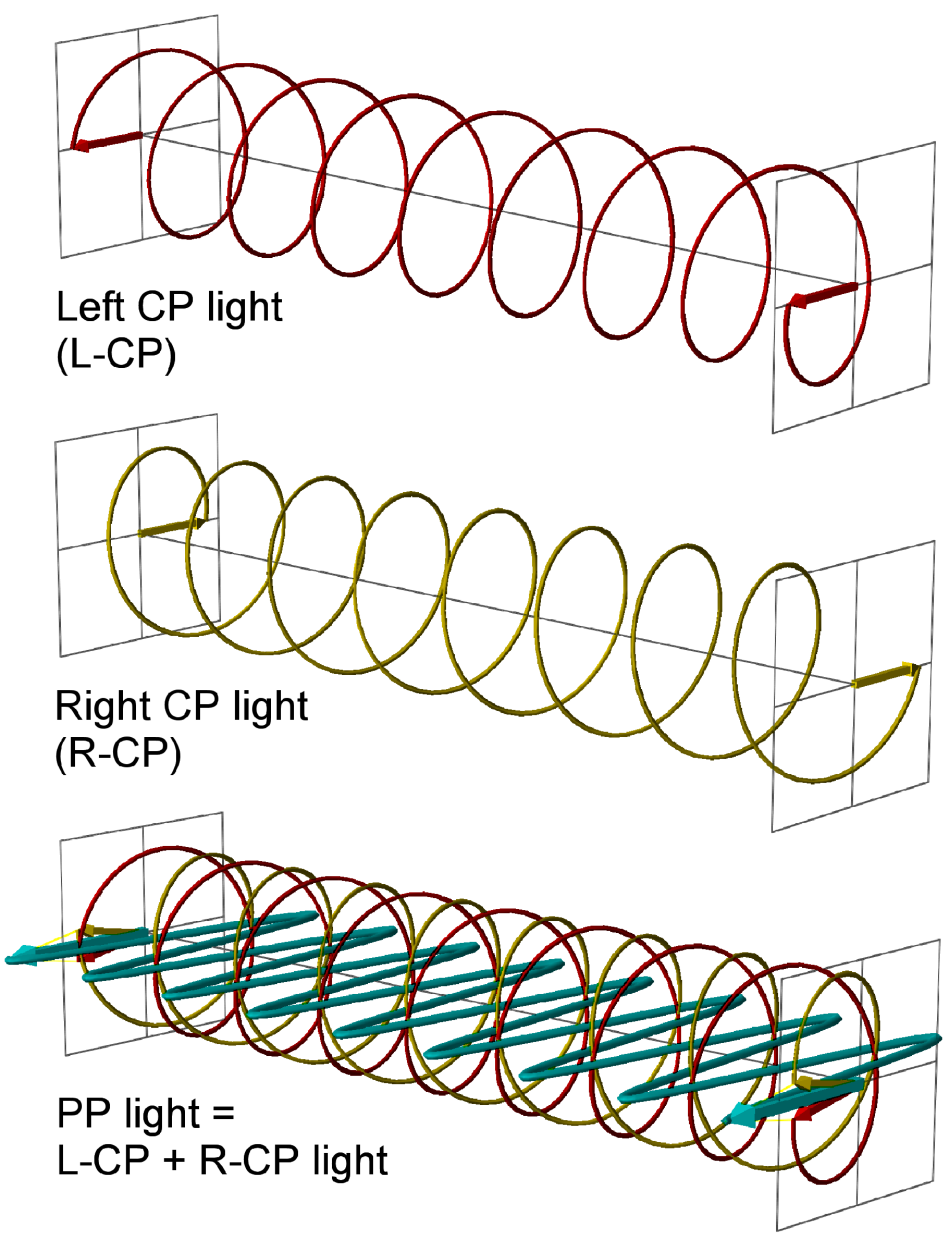
Plane polarized light (PP, cyan) as the sum of the left and right circularly polarized light (L-CP, red, and R-CP, yellow). L-CP is defined as such as an observer far from the source (on the right side of the figure) will see a vector describing an anti-clockwise circle.

Many biological molecules are chiral and chromophoric among which the most important examples include proteins and nucleic acids. Moreover, the chiral constituents of natural biopolymers are homochiral, e.g., (almost) exclusively L-amino acids and D-sugars are found. Among other properties, chiral chromophoric molecules absorb left circularly polarized light (L-CP light) differently from right circularly polarized light (R-CP light). L-CP and R-CP light can be seen as the two components, rotating in opposite directions (anti-clockwise vs clockwise), of plane polarized light (PP, Figure 1). Thus, the use of circularly polarized light has led to the development of several spectroscopical methods for the study of chiral non-racemic molecules, including biopolymers [1–4]. Particularly important applications of these methods are found in structural studies of biomacromolecules [3]. For instance electronic circular dichroism (ECD), the most commonly used method, is indispensable in the structural studies of proteins and also intensively used in the characterization of the helical structure of DNA and RNA [5]. As nucleic acids are characterized by a dominant helical chirality and exhibit a rather small set of secondary structures, each characterized by a different polarization spectroscopy signature, they are convenient targets to monitor structural changes induced by outer stimuli. Linear dichroism (LD) is another type of polarization spectroscopy which does not require a chiral sample but rather an oriented one. It is based on the differential absorption of light polarized either parallel or perpendicular to a certain axis of orientation. This is also applicable to biomacromolecules such as nucleic acids, whose helices are normally elongated in a single direction [2]. In this context one of the most common approaches is to monitor the changes in the ECD or LD spectrum upon binding of a ligand to DNA or RNA [6].

Non-covalent interactions of small molecules (ligands) with DNA and RNA are of paramount interest because many biological processes, drugs and biochemical tools/probes rely on them [7–8]. Due to the possibly multifaceted nature of the complex formed between a small-molecule ligand and a large receptor (DNA/RNA), several complementary methods are needed for the accurate characterization of their interactions. Although the design and performance of experiments used for DNA/ligand interaction studies are common to the various techniques described here (and also to standard UV–vis, IR or fluorescence spectroscopy), there are several important differences related to the sensitivity of the methods, possible artifacts and the interpretation of the obtained results. Since some of the significant facts are often neglected, the obtained results may fail to give reliable information or are misinterpreted.

With the hope that a detailed description of the experimental conditions and general rules for the interpretation of results will lead to more extensive applications and higher accuracy of the results, several reviews and tutorials were written in the past. For instance, a tutorial focused on circular dichroism as a tool for studies of non-covalent ligand/DNA interactions [9], provided a step-by-step protocol to the measurement. Very recently, a similar tutorial was published for LD experiments [10], and also a general outline on how to interpret CD and LD results with respect to the most common ligand/DNA binding modes was summarized on a more comparative basis [6]. Within the last decades, complementary methods to ECD also were developed, for instance, vibrational CD (VCD) was successfully applied to investigate DNA/ligand interactions [11]. Furthermore, fluorescence detected circular dichroism (FDCD) combines the advantages of both CD and fluorescence emission technique, which is ideal for the selective study of DNA ligands that strongly change fluorescence upon binding [3]. In a sense complementary to FDCD, also circularly polarized luminescence (CPL) is a chiroptical emission technique which has been employed in the same context for the first time recently [12].

**Figure 2:**
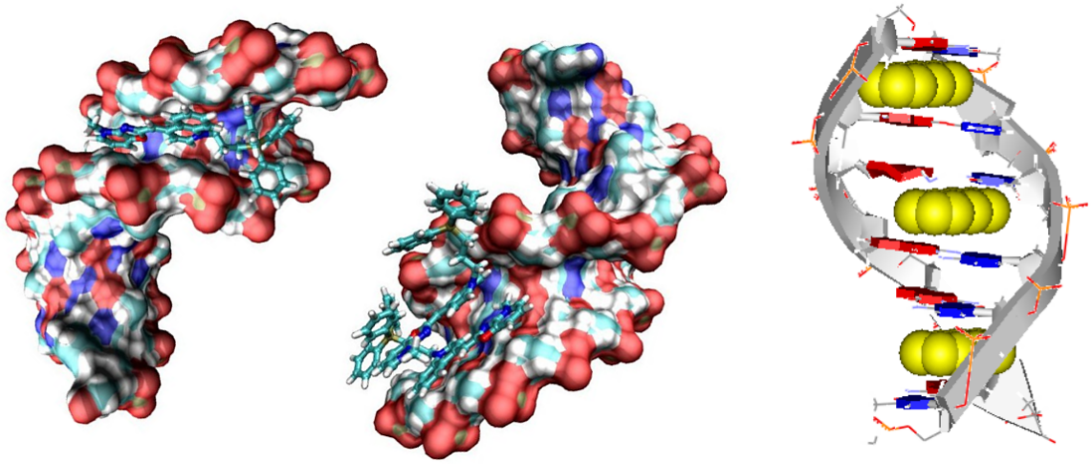
Schematic representation of ligand binding modes to DNA or RNA (left to right: DNA minor groove binding, minor groove aggregation, intercalation), which can be distinguished by polarization spectroscopy methods.

The aim of this review is to summarize in one tutorial all required information and practical advice for performing experiments with the most common polarized spectroscopy methods: ECD, LD, VCD, FDCD and CPL with the goal of an accurate determination of interactions of small molecules with nucleic acids. Among the methods discussed, primary focus will be centered on the most frequently employed techniques, ECD and LD. Moreover, we will elaborate the interpretation of results not only for the most common DNA, RNA/ligand binding modes (intercalation, groove binding) but also for increasingly appearing ligand aggregates binding to polynucleotides (Figure 2). Many naturally occurring small molecules owe different biological activities due to aggregation, for instance, the close analogs netropsin (the single molecule in the DNA minor groove) and distamycin (the dimer in DNA minor groove) [13]. In addition, we will discuss the newest possibilities of computational analyses of the results as an outreach from the currently used empirical rules [6] for the determination of the ligand binding mode.

### 2. General aspects

ECD and LD are based on the phenomenon of light absorption by one or more chromophores in the UV–vis range of the electromagnetic spectrum, where electronic transitions occur. The application of these polarization spectroscopy methods for the study of a complex between a small molecule (ligand) and DNA or RNA can generally be divided into two wavelength ranges:

a) monitoring changes in the wavelength range where DNA and RNA absorb light (λ = 200–300 nm) and thus possess intrinsic spectra. Changes in the intrinsic spectral properties of DNA or RNA can often be correlated to a specific change in the secondary structure of the polynucleotide (see chapter 2.1.). However, if a ligand’s chromophore also absorbs in this range, the deconvolution of all contributions is not trivial (see interpretation of results in the chapters of the corresponding methods);

b) monitoring changes in polarization spectra at λ > 300 nm for ligands that have corresponding chromophores. This is the most common used approach as it monitors changes of only one species involved in the complex formation and thus the interpretation of results is simple and straightforward.

However, to design the polarization spectroscopy experiment accurately, it is essential that solutions of both the polynucleotide and the ligand are adequately prepared and characterized. For that reason, a short description of the most important issues in sample preparation is summarized in chapters 2.2 and 2.3.

#### 2.1. Relation between DNA or RNA secondary structure and polarization spectroscopy

**Scheme 1:**
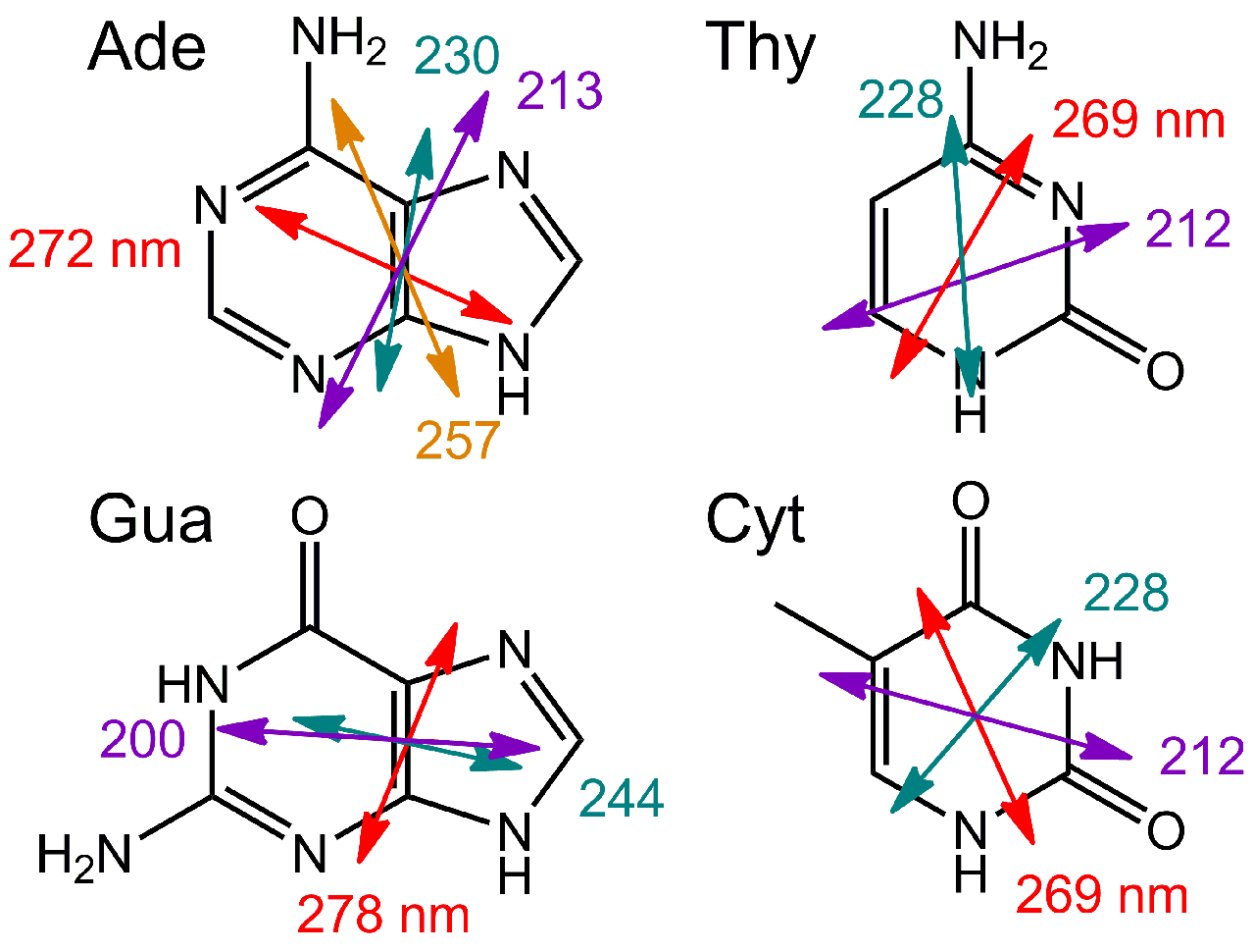
Principal electronic transitions of nucleobases (uracil is similar to thymine, Thy). The arrows depict the polarization direction of each transition, and the length is roughly proportional to the relative intensity.

**Figure 3:**
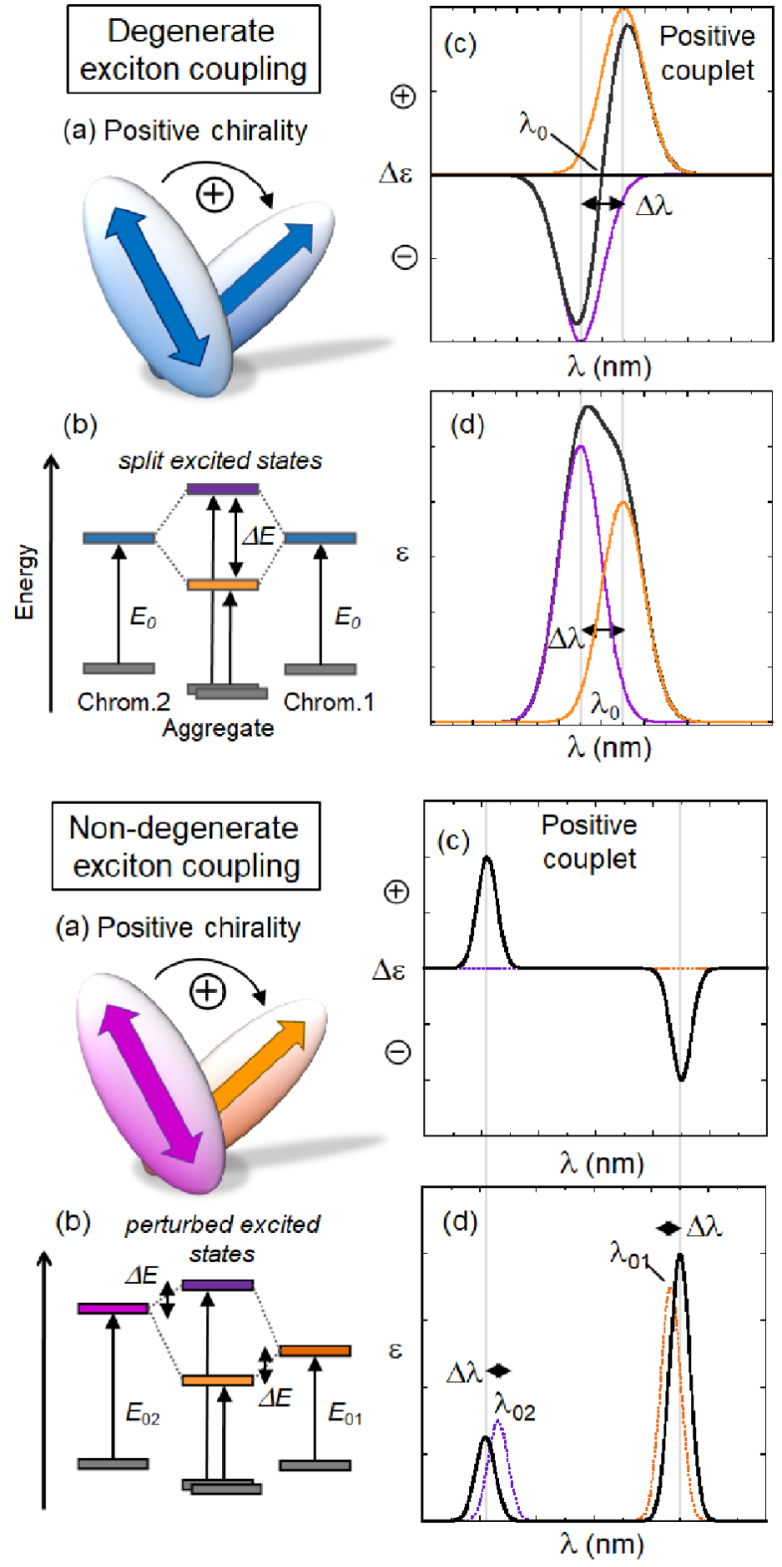
Exciton coupling mechanism in its degenerate (top) and non-degenerate version (bottom). a) Chirality defined by transition moments located on the two chromophores. A positive chirality corresponds to a clockwise direction when rotating the transition dipole in the front onto that in the back. b) Splitting of excited states due to exciton coupling. Notice the stronger effect in the degenerate case. c) ECD couplet originating from exciton coupling. The sign of the couplet (i.e., of its long-wavelength branch) is the same of the chirality (exciton chirality rule). In the degenerate case, the couplet is centered in correspondence of the chromophore transition at λ_0_. In the non-degenerate case, each band is localized close to one chromophore transition (λ_01_ and λ_02_). d) Absorption spectra originating from exciton coupling. The wavelength splitting Δλ is related to the energy splitting Δ*E*.

Nucleobases are achiral but nucleoside and nucleotide derivatives are optically active. The π→π* transitions of the bases (Scheme 1) contribute to the electronic circular dichroism mostly as a result of the chiral perturbation exerted by the sugar moiety. The ECD signals of single nucleosides/nucleotides are, accordingly, quite small. Coupling into polynucleotides, particularly in the double-stranded helix of DNA or RNA, introduces helical chirality, whereby the helical axis is almost perpendicular to the aromatic base-pair plane. In this situation, the ECD changes dramatically as a consequence of the so-called coupled oscillator or exciton coupling mechanism between the various π→π* transitions of regularly arranged chromophores [14] (Figure 3, top).

**Figure 4:**
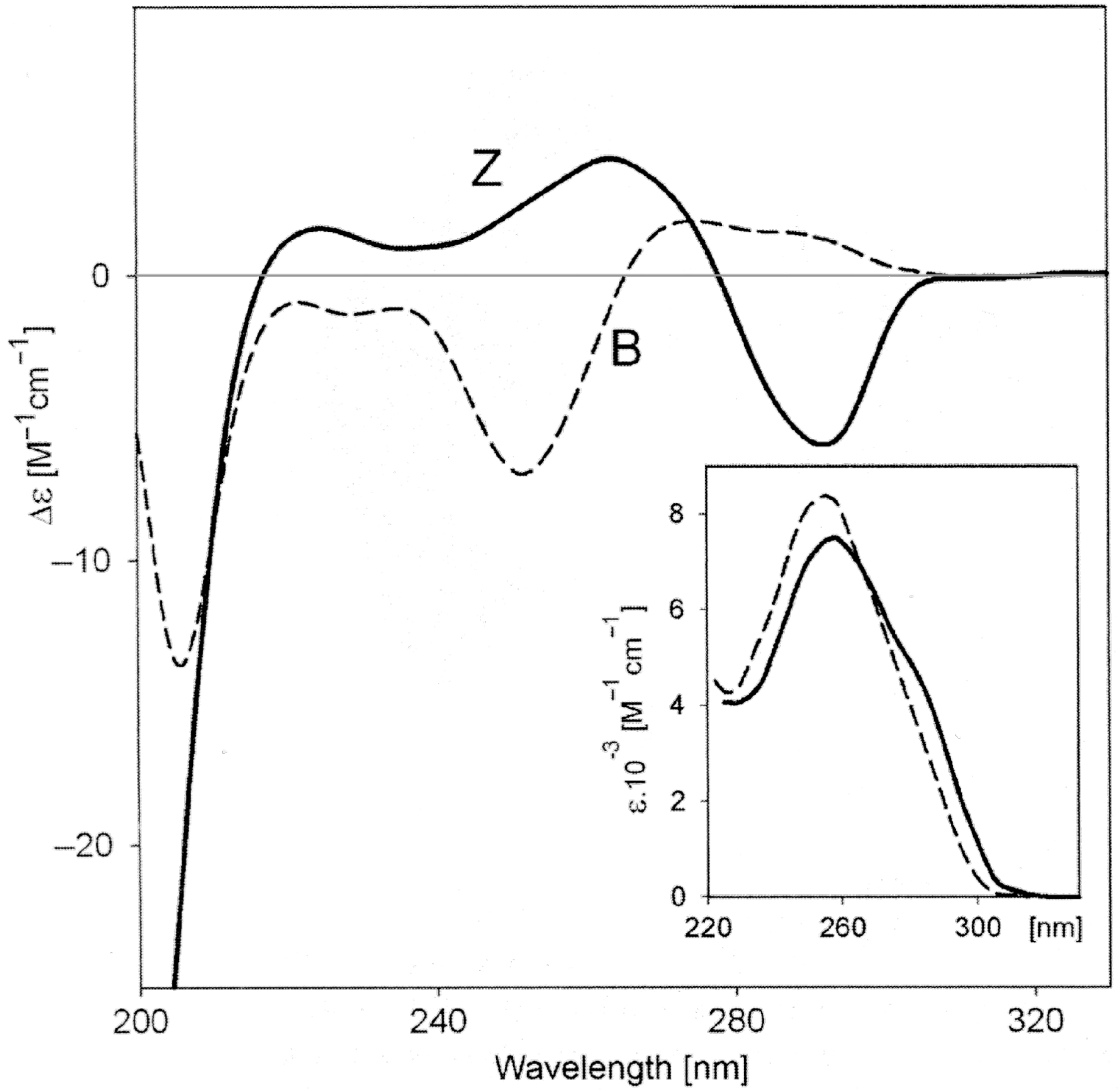
ECD and UV (inset) spectra of poly(dG-dC)–poly(dG-dC) in the B and Z-form, obtained in 59% and 67% aqueous trifluoroethanol, respectively. Adapted from [15].

Chiroptical properties and ECD spectra of particular DNA or RNA sequences are therefore strongly dependent on the polynucleotide secondary structure [15], at variance to the common UV–vis spectra of the same samples (Figure 4). Of course, this fact has very important practical applications in monitoring the polynucleotide structure change caused by outer stimuli like ligand binding, pH changes, melting, etc. Intriguingly, most of the single-stranded (ss) polynucleotides, if long enough, also show some extent of secondary chiral organization, thus also allowing a monitoring by ECD spectroscopy [16–17]. The non-degenerate coupled oscillator mechanism is also responsible for generating ECD signals in correspondence with electronic transitions of achiral chromophores surrounded by a chiral environment. The most relevant example here is that of an achiral ligand bound to a nucleic acid, yielding a so-called induced circular dichroism (ICD) whose sign and magnitude are determined by the binding geometry. In this case, the exciton coupling is said to be non-degenerate (Figure 3, bottom). It must be stressed that the binding also determines a change in the standard absorption bands of the ligand, which can be similarly monitored to study the interaction [18].

Detailed information about various DNA/RNA structures and their ECD spectra is out of the scope of this review. A specific example concerning guanine quadruplexes is discussed in chapter 3.

#### 2.2. DNA or RNA stock solution preparation

Here we discuss in detail only polynucleotides with lengths of more than 100 base pairs because oligonucleotides with known composition are much easier to dissolve, to check their structural properties and to determine exact concentrations. Also, short oligonucleotides can fail in representing a biologically significant structural model, because of heterogeneous binding sites due to the “capping” effect, whereby a ligand can bind similarly to the end base pairs and to a binding site along the helix [19].

Commercially available nucleic acids are usually sold as lyophilized white fibers and should be stored as defined by the producer before dissolving. Most of the nucleic acids are available as sodium salts and a variety of different sequences is available. *Calf thymus* DNA is the most commonly used DNA extracted from *calf thymus* tissue which consists of 41.9 mol % of G–C and 58.1 mol % A–T base pairs. Other natural DNAs with different base-pair ratios are not that easily available. However, a diversity of synthetic polynucleotides is commercially available, such as double-stranded nucleic acids with alternating or homo-base sequences as well as single-stranded homo-polynucleotides. Such synthetic DNA or RNA have well-defined structural properties and therefore are recommended for studies of small molecules targeting structural DNA or RNA selectivity.

All polynucleotides should be dissolved exactly as defined by the producer, usually in a buffer of defined ionic strength. A common mistake is disregarding the proposed procedure by using too low ionic strengths, which severely impacts the double-stranded helix folding and stability, and consequently strongly influences the corresponding ECD spectrum. Another common mistake with a significant impact on further experiments is preparing a too highly concentrated polynucleotide stock solution. Particularly, guanine-rich polynucleotides are prone to gelation and consequently to formation of non-homogeneous solutions. Thus, an optimal concentration for the stock solution of polynucleotides would be about 0.01 M. It is calculated as nucleobase/mol by using the molar extinction coefficient at the maximum of the wavelength absorbance (as defined by the supplier). In addition, dissolving polynucleotides is not an instantaneous process and usually takes several hours at room temperature. When DNA or RNA stock solutions are prepared, collecting the absorption and ECD spectra is the best way to check their quality.

Commonly, solutions of synthetic polynucleotides are stored at −20 °C and used without any further purification. However, DNAs isolated from natural resources (e.g., *ct*-DNA as the most common) are quite often exceptionally long. Therefore, upon soaking the dry fiber in the buffer, a very viscous solution of approximate 0.01 M concentration is obtained, that is hardly applicable for accurate titration experiments. In this case, it is advisable to sonicate the DNA solutions by sonication tips that are common in the biological laboratory, and a treatment for several times in 5-second periods affords a much less viscous solution and much shorter (about 100 base pairs) rod-like B-helical DNA fragments. There are several reasons why sonication of DNA isolated from natural resources is essential: the titrations with very viscous or even gelating non-sonicated DNA solutions are not accurate for practical reasons. The accuracy of automatic pipets and standard tips is designed for aqueous solutions of viscosities similar to that of pure water and, in addition, not all potential small-molecule binding sites on DNA-supercoiled fragments are accessible to the small molecule, therefore leading to erroneous site-size evaluation.

#### 2.3. Ligand solution preparation and characterization

Preferably, the ligand should be dissolved in aqueous solution at a concentration of about 0.001 M. The prepared solution should be clear and homogeneous with no visible precipitation or opalescence. If the ligand is poorly soluble in water, other solvents can be used, but some of them may interfere by absorbing light and raising the wavelength cut-off, as well as by interacting to some extent with DNA or RNA structure. The most common and efficient solvent for this purpose is DMSO. It does not interfere significantly with DNA or RNA properties up to 0.1% v/v [20], while at higher DMSO quantities, its impact on ECD spectra of DNA or RNA should be corrected for. However, even at this small ratio mentioned above, DMSO significantly absorbs light at <240 nm, hampering an accurate collection of data in the short-wavelength region.

It is essential to check the dependence of the ligand’s UV–vis spectrum on its concentration in the experimental conditions foreseen for further experiments. If the light absorbance is proportional to concentration, i.e., the Beer–Lambert law is fully respected, there is no intramolecular aggregation of the ligand. However, a non-linear response, usually hypochromic (i.e., a negative deviation from the Beer–Lambert law), supports the formation of ligand aggregates. If it is not possible to further dilute the sample to be used for polarization spectroscopy experiments, ligand solutions should be treated as a mixture of ligand plus its aggregates in thermodynamic equilibrium and analyzed accordingly. Similar to the absorption spectra for chiral ligands, ECD spectra at different concentrations can be collected, taking into account that the total absorbance of the sample is compatible with the sensitivity of the instrument. Again, aggregating ligands could form chiral aggregates with a characteristic exciton-coupled CD spectrum, which is by the way diagnostic for the supramolecular chirality [21–22].

Particular attention should be a paid to the UV–vis and ECD spectrum shapes and baselines as well as to an opalescence of the solution in the cuvette. An apparent positive drift of the baseline during ligand concentration increase in the cuvette, usually best seen in the long-wavelength range of the spectrum, is a clear evidence of precipitation or colloid/aggregate formation in the cuvette, which completely hampers further experiments.

The characterization of the thermal stability of the ligand solution by collecting it’s UV–vis spectrum and if applicable, ECD spectrum at various temperatures, can give useful information about intra- or intermolecular interactions of a ligand. However, an ECD spectrum depends on the temperature in several ways other than affecting aggregation, for example by changing conformational populations. Therefore, a variable-temperature ECD spectrum is often not easy to interpret. Only after a detailed characterization of the ligand solution, experiments with polynucleotides can be performed by a series of different techniques described in following sections.

The electronic circular dichroism (ECD) will be discussed first as the most common technique with the least pitfalls. Thus it is most often appropriate for the characterization of the systems of interest. Then, LD follows as a complementary method used to reveal the mutual orientation of the ligand chromophore with respect to the DNA/RNA helical axis. The other techniques mentioned in the Introduction (VCD, FDCD, and CPL) will be treated more shortly because of their relatively less widespread use in the context of interest. The particular aspects of data analysis and interpretation will be discussed for each method, while general aspects including computational approaches and comparison of the obtained data from several methods will be summarized in the last chapter.

### 3. Electronic circular dichroism (ECD)

**Figure 5:**
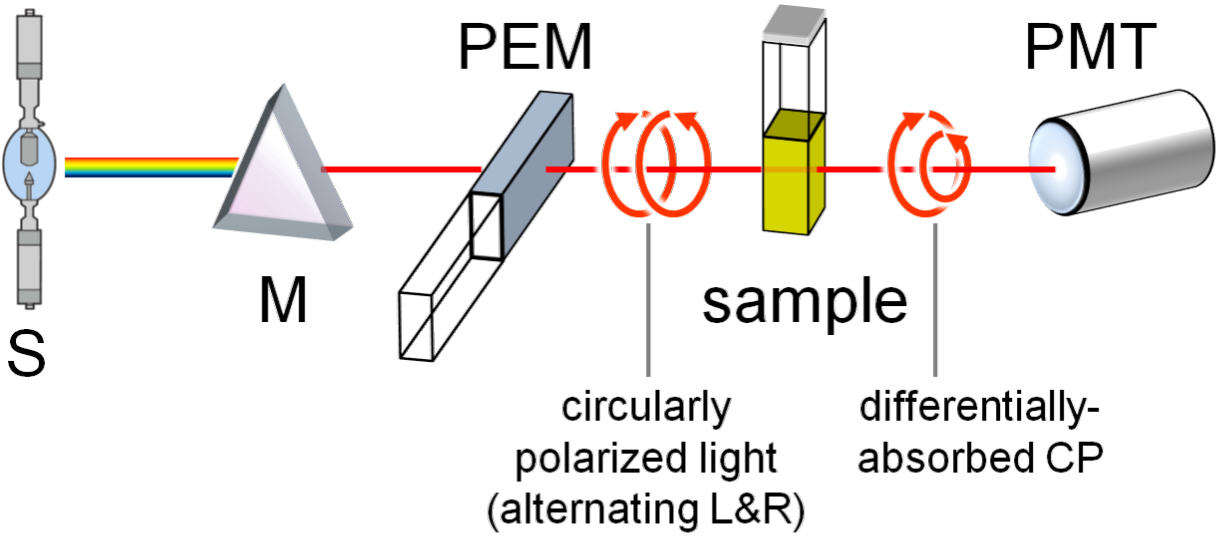
Schematic illustration of an ECD instrument. Legend: S, source; M, monochromator (wavelength selector); PEM, photoelastic modulator producing L-CP and R-CP light alternatively; PMT, photomultiplier (detector). A basic VCD instrument has a similar design, except that the monochromator is replaced by an interferometer for Fourier transform.

Electronic circular dichroism (ECD) is the difference in absorption between left and right circularly polarized light (Figure 5). It provides information about the chiral species in solution which absorb light in the UV–vis range, due to transitions from the electronic ground state to one or more excited states. ECD is one of the most sensitive spectroscopic techniques for probing changes in the DNA or RNA binding mode of a ligand as a function of concentration and/or mixing ratios [3,5–6,9,18]. However, ECD relies on the difference in absorption between left and right circularly polarized light which makes it at least two orders of magnitude less sensitive than absorption spectroscopy [1,9].

**Figure 6:**
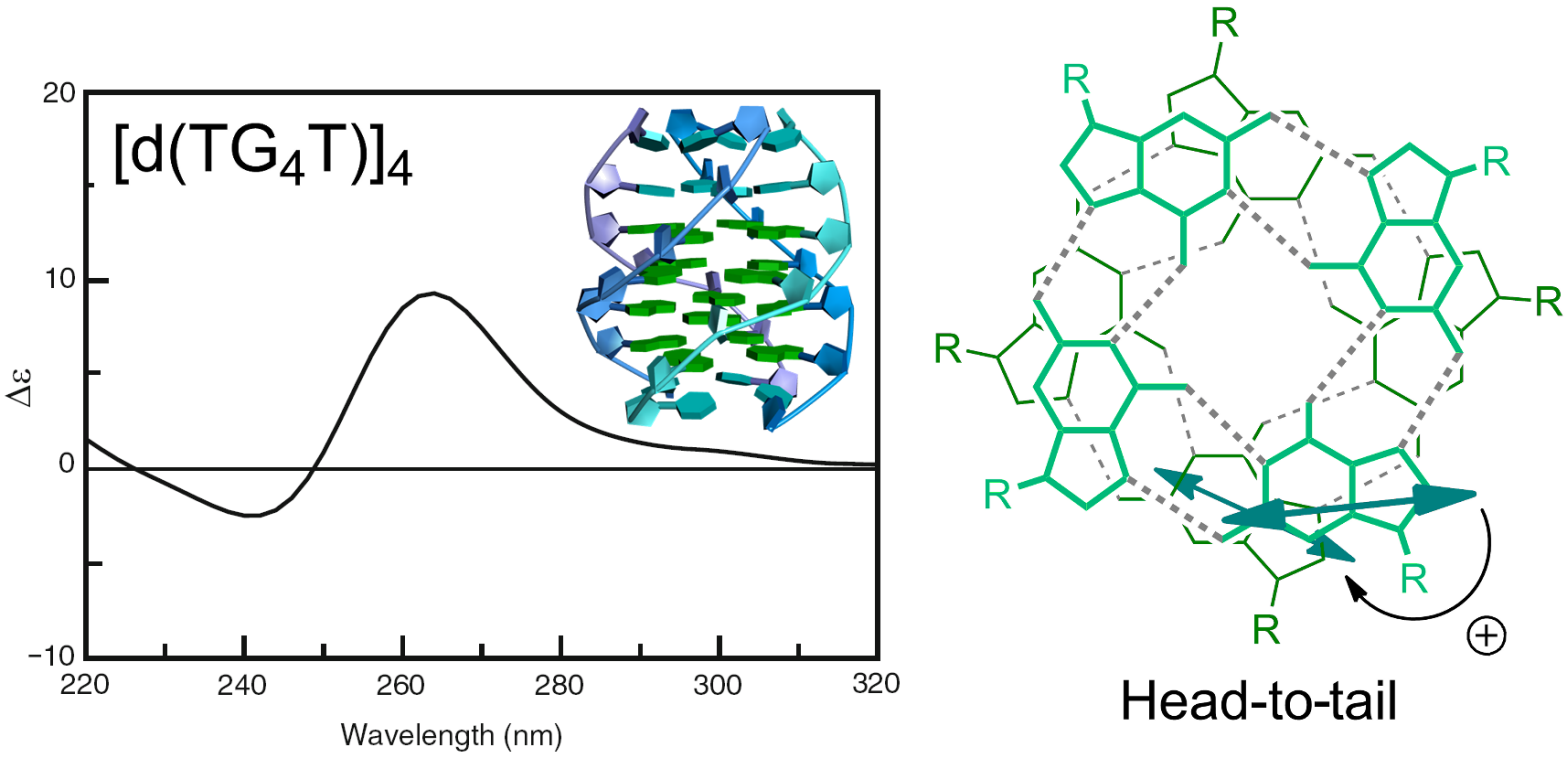
Exciton coupling in the G-quadruplex [d(TG_4_T)]_4_. For this sequence, the quadruplex is formed by four parallel strands and the quartets face each other in a head-to-tail arrangement. Reprinted in parts with permission from [22] to G.P. as an author of the article, copyright 2014, The Royal Society of Chemistry.

ECD spectroscopy can give significant structural information based on electric and magnetic transition moments of adjacent chromophores and their mutual orientation. This is especially true in the presence of strong chromophores interacting through the coupled oscillator mechanism mentioned above. Consequently, based on the organization of DNA/RNA chromophores (base pairs) in a helical structure, ECD is primarily sensitive to the secondary structure of various nucleic acids and provides characteristic ECD spectra of nucleic acids as a result of base sequence and experimental conditions (see chapter 2.1.). As an example of how the exciton coupling mechanism determines the ECD spectra of polynucleotides, Figure 6 reports the case of guanine quadruplexes (G-quadruplexes) which are easy to analyze because of the presence of a single type of nucleobase and a rigid structure [23]. The ECD spectrum is dominated by the degenerate exciton coupling between the major transition of guanosine around 245 nm; the transition is long-axis polarized (Scheme 1) and, for a right-handed quadruplex arrangement, it defines a positive chirality between stacked guanines, which yields a positive couplet. Even in this sample case, ECD is, however, also affected by other contributions, e.g., the non-degenerate coupling between different guanosine transitions, long-range couplings between distant guanosines, etc.

More interestingly for our purpose, ECD is a useful method for probing conformational changes of nucleic acid upon ligand binding. Moreover, as also mentioned in chapter 2.1., achiral ligands upon binding to DNA or RNA can eventually acquire an induced CD (ICD) spectrum, especially when their transition moments are uniformly oriented with respect to the DNA/RNA binding site, which could give useful information about modes of interaction and binding geometry, as will be discussed below.

ECD spectroscopy shows several advantages for investigating DNA, RNA and their complexes with ligands with respect to other spectroscopic techniques (non-polarized spectroscopies, NMR, X-ray single crystal diffraction) [6,9]:

- The experiments are technically easy and comparatively quick to perform.
- The equipment for sample holding is broadly available, common to any other solution spectroscopy methods. Standard 1 cm quartz cuvettes for absorption and/or fluorimetric titrations can be used.
- The experiments are performed in solution (easily mimicking physiological conditions) with a concentration lower than that needed for NMR measurements. Moreover, ECD is a “fast” spectroscopy so that the typical problems relative to the NMR timescale are not encountered.
- Crystalline material is not required as for the X-ray single crystal diffraction technique.
- ECD spectroscopy is sensitive to a mutual orientation between the DNA/RNA axis and the orientation of the ligand transition moments. Thus it quite often yields detailed structural information about the studied complex. This is the most important advantage with respect to the use of non-polarization spectroscopy, e.g., absorption spectra.

However, some characteristics of the ECD instruments limit the experimental conditions. The most important limitations are: a) the total absorbance of samples at a chosen signal through the complete experiment should ideally be below 1 a.u. (higher absorbance values up to 1.8 a.u. may still lead to sufficiently accurate spectra, especially with last-generation instruments, but should be checked for reproducibility); b) induced (I)CD bands could have poor signal-to-noise ratios for, e.g., intercalators or for any weak ICD signals (the sensitivity of standard ECD instruments is *g* ≈ 10^−5^, where *g* = ΔAbs/Abs = Δε/ε); the common approach of collecting a number of scans to improve the signal-to-noise ratio is time consuming. Thus, for the collection of titration data necessary to obtain an accurate binding isotherm (10–90% of complex formed, an excess of DNA binding sites over ligand), the application of the ECD method may be limited by available material and long times. This is particularly valid if the studied system and its binding constant are unknown and repetition/redesign of the experiment is necessary.

Recently, Garbett, Ragazzon and Chaires [9] summarized a general protocol for the use of ECD for the simultaneous determination of the binding mode and binding affinity of ligand/DNA complexes. In the light of the limitations listed above, here we would present an alternative approach, whereby an unknown DNA (or RNA)/ligand system is characterized primarily by a set of common methods (UV–vis spectroscopy or fluorimetric titrations processed by non-linear fitting procedures [9,24], thermal denaturation experiments [25]). According to these results, a set of several ECD spectra is designed particularly for the target of interest. For instance, a) characterization of single molecule binding would be studied at large excess (10 to 50-fold) of DNA/RNA over ligand; b) ligand aggregation within DNA/RNA would be studied at excess of ligand over DNA/RNA; c) kinetics of binding would be studied at different temperatures and instrument response times; d) competition experiments between two ligands aiming for the same binding site would require a specific design; and so on.

#### 3.1. Practical information

**Cuvettes:** quartz, preferably high-quality manufactured with precisely parallel walls (e.g., fluorimetric cuvettes) and minimal residual strain. Cylindrical cells are usually recommended for ECD, but the standard cuvettes may also be used, if they pass the following test. Check of quality: collect the ECD spectrum of the buffer, rotate the cuvette 180° about its vertical axis and collect the spectrum again. The obtained two spectra should overlap well. If not, and especially in the presence of bands with intensities > 1 mdeg and opposite sign for the two measurements, the cuvette should be replaced. A broad range of path lengths is available (0.1 mm–5 cm) to ensure the optimal conditions of the total sample absorbance (about 0.8 Abs is recommended, with 1.5 Abs as upper limit). Commonly 1 cm, 3 mL cuvettes are used, filled with a sufficient amount of the sample in such way that the light beam does not pass close to the meniscus. Narrow cuvettes (widths < 0.5 cm) should have black-masked side-walls to prevent light beam reflection and absorption flattening artifacts [26]. The same cuvette should be used for all the measurements of one system (titration). A good cleaning of the cuvettes is essential (see producer manual or [9]), particularly for ligands that adhere to glass (e.g., porphyrins). For particularly adhering ligands, whose ICD spectra are measured in the vis range (>360 nm), the application of disposable plastic cuvettes can solve or at least minimize the problem. However, for dyes that strongly adhere to cuvette walls the collected chiroptical spectrum does not correspond to conditions in homogeneous solution and thus cannot be interpreted according to here given rules.

**Buffers:** DNA and RNA require specific buffers and ionic strengths to be reliably folded into their native secondary structure. Significantly lowering the ionic strength before or during the experiment (i.e., upon dilution by ligand solution addition) will not only impair buffering capacity but also will influence the DNA/RNA secondary structure and consequently change the corresponding ECD bands. Commonly, the experiments should be performed at pH 5–8 and ionic strength >1 mM.

**Instrumental conditions:** Instrument parameters differ among the instrument producers; here we will suggest those of Jasco J-810, but other instruments have corresponding ones. The parameters to control are: wavelength range, scan rate, bandwidth, averaging time and number of scans.

Wavelength range: Start: 220 nm (at lower wavelengths the total absorption of samples is high and requires specific conditions). End: maximum absorption of sample + 50 nm (the additional wavelength range is required to monitor if the baseline beyond the absorption region of the sample is flat after spectrum subtraction).

It is compulsory to monitor in parallel the two channels corresponding to ECD and HT (related to absorbance) signals. The ECD channel is often reported in mdeg, related to ellipticity. To convert mdeg in absorbance units, the following formula holds: θ (mdeg) = ΔA/33000.

In biopolymers fields, the molar ellipticity [θ] is still used, defined as [θ] = (θ/*lc*) where *l* is the path length in cm and *c* the molar concentration. We, however, recommend its conversion in molar circular dichroism: Δε = ΔA/*lc* = [θ]/3300.

On the other hand, if the ECD data are used to monitor ligand binding, they can be directly plotted and interpolated in mdeg. In general, the slower the collection of data and the larger the number of scans are, the better is the quality of final data. However, it is better to average a number of faster scans than collect one scan very slowly. A more detailed analysis of parameters, which are necessary for the collection of high-quality data for binding isotherms is given in a specialized review [9]. Here we propose parameters allowing for the collection of one spectrum within a reasonable time and an acceptable quality. The following settings are recommended: Scanning speed: 50–100 nm/min (a faster speed is applicable for ECD spectra with wide bands); response (time constant): 1 s; bandwidth: 1 nm; accumulation of scans, which are averaged in one spectrum: 4, 8 or 16.

Of course, temperature accuracy is crucial in experiments involving DNA/RNA. Modern ECD instruments are equipped with a dedicated Peltier apparatus, which needs to be adjusted to the desired temperature.

#### 3.2. Practical binding experiment/step by step procedure

- Put 2 mL of the buffer solution into a 1 cm path length cuvette (with a total volume of 3 mL) and record the spectrum of the buffer. The ECD spectrum of the buffer will not be zero, so the buffer background spectrum should be subtracted after each addition of DNA and sample. Remember the orientation of the cuvette in the holder and maintain it throughout the experiment.

- Add an aliquot of DNA stock solution to the cuvette to get *c*(polynucleotide) = 10–40 µM in the cuvette. Subtract the buffer spectrum from the DNA spectrum. The baseline in the range λ > 300 nm should be zero. If not, check for turbidity of the solution or other causes referred in chapter 2.

- Then, add aliquots of ligand stock solution (preferably at mM concentration) into the cuvette to cover the ratio r_[ligand]/[polynucleotide]_ = 0.1–1 in 0.1 step size (within this range all major binding events shown in Figure 2 are usually detectable and can be subjected to detailed analysis). The incubation time prior to the collection of the spectrum depends on the kinetics of binding, determined previously by other methods (UV–vis or fluorimetric titration). After each addition, the buffer background spectrum should be subtracted.

It takes about 10–20 min to collect one spectrum by multiple accumulations. During that time, the previously recorded spectra can be processed and compared to follow in real time the evolution of spectra. Any deviation from the baseline in the range where the studied ligand/DNA do not absorb light should be immediately inspected for turbidity (chapter 2). Furthermore, the Abs channel should be continuously monitored, taking care that the total absorbance of the sample does not exceed 1.5 or the limit suggested by the vendor.

#### 3.3. Interpretation of results

**3.3.1. Wavelength range < 300 nm:** If the ligand does not absorb light within this range, the changes in the intrinsic ECD spectrum of the DNA and RNA can be correlated with a change in the secondary structure due to ligand binding. For instance, a significant decrease in the ECD spectrum over the whole 200–300 nm range indicates a disruption of helical chirality by intercalation or severe kinking of the helix by sterically demanding groove binders. At variance, if the ECD spectrum of the DNA and RNA does not change significantly, the biopolymer helical structure is preserved, suggesting a ligand groove binding, outer surface binding, or no/weak binding.

If a ligand does absorb light within this range, the observed changes cannot be unambiguously attributed to the DNA/RNA, because the ICD of the ligand (which is in principle unpredictable in intensity and sign), will combine with the intrinsic ECD spectrum of the polynucleotide. In special cases of poorly organized ss-polynucleotides, the intensity of the polynucleotide ECD bands may significantly increase while preserving the ECD band fingerprint. If so, one can presume a strong increase of polynucleotide helicity [27].

**3.3.2. Wavelength range > 300 nm:** In this range DNA and RNA do not absorb light. Thus, all ECD signals can be attributed to the ligand solely. The most straightforward case occurs with achiral ligands, whereby ligand binding to DNA/RNA results in an induced (I)CD spectrum.

If the ligand is chiral and has an intrinsic ECD spectrum (see chapter 2.3., characterization), the difference in the ECD spectrum caused by ligand binding is obtained by subtracting the intrinsic spectrum. However, it should be taken into account that the observed change in the ECD spectrum can have several origins: a) a change of the ligand’s inherent chirality due to the structural changes caused by binding; b) an induced (I)CD as a result of ligand insertion into chiral binding sites; c) exciton coupling between multiple aggregated ligand molecules.

The appearance of one or more isodichroic points (the CD equivalent of isosbestic points) during ECD titration suggests the formation of one dominant type of DNA or RNA/ligand complex. Usually, a shift of isodichroic points can be observed during the titration, if it is pushed up to a large excess of ligand over DNA or RNA. This indicates that another, usually less preferred binding mode takes place. Isodichroic points can be found in the DNA absorbing range as well as in a range >300 nm where the ligand absorbs.

**Figure 7:**
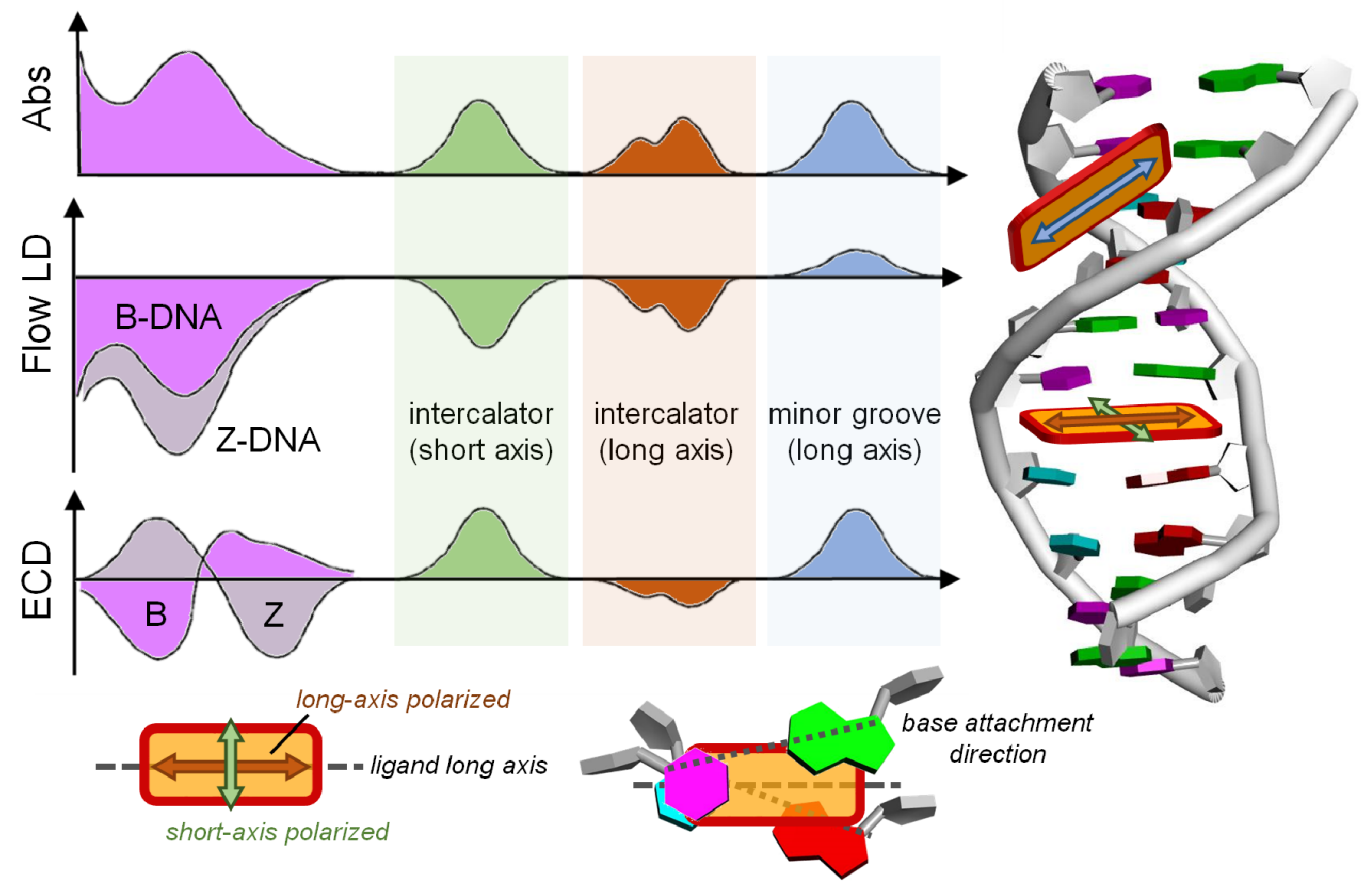
Empirical analysis of ECD and flow LD spectra to establish the preferred orientation of a ligand bound to DNA. We define the transition moment of the ligand oriented “parallel” to the long axis of adjacent base pairs, when it is halfway between the closest base attachment directions (bottom right panel). The LD is sensitive to the arrangement of the dye transition moment with respect to the helix axis, and to the overall orientation of the DNA double helix with respect to the flow direction (see Figure 9, below); Z-DNA is easier to orient than B-DNA because of a more extended elongation. ECD in the region <300 nm reflects the DNA conformation, in particular the mutual arrangement between nucleobases, which is different for right-handed B and left-handed Z-DNA. In the region >300 nm, the ligand ICD depends on the arrangement of the ligand chromophore with respect to the nucleobases. The sign of ICD is opposite for mutually orthogonal transition moments (long vs short axis) allied with an intercalated chromophore.

In the experimental conditions of one dominant binding mode (excess of DNA/RNA binding sites, clear-cut isodichroic points), there is a restricted number of possible outcomes for an achiral ligand which can be interpreted in a qualitative way to afford information about the preferred binding mode (Figure 7). The following conclusions stem from a large collection of experimental data and must be regarded as empirical [2,9]. However, they have also been substantiated by some of the theoretical approaches which will be mentioned in chapter 7.2.

**3.3.3. Weak ICD (intensity of ICD band several times lower than the ECD band of DNA/RNA):** a) Negative sign, non-linear relation of ICD intensity to ratio r_[ligand]/[DNA]_ approaching saturation at about r = 0.2–0.3. This is a strong indication of intercalative binding, with the transition moment of the ligand oriented “parallel” to the long axis of adjacent base pairs (Figure 7, brown hue and bottom-right panel). It should be additionally supported by: i) a red-shift of the ligand absorption band; ii) at least moderate thermal stabilization of ds-polynucleotide; and iii) at least 10 µM binding constant (at common conditions, pH 5–8, *I* = 0.05–0.1, rt).

b) Positive sign, non-linear relation of ICD intensity to ratio r_[ligand]/[DNA]_ approaching saturation at about r = 0.2–0.3. This indicates either groove binding with a loose orientation of the ligand with respect to the DNA axis or intercalative binding with the transition moment of the ligand oriented perpendicular to the long axis of adjacent base pairs (Figure 7, green hue). Additional experiments, preferably NMR are needed.

c) Negligible ICD intensity within signal-to-noise ratio, although other methods indicate a strong binding. Most likely intercalation takes place with the transition moment of ligand oriented at an angle to the long axis of adjacent base pairs, which happens to cancel positive and negative contribution. This could also be an indication of a ligand binding on the outer DNA/RNA surface through electrostatic interactions with the phosphate backbone. However, that is plausible only for highly positive-charged ligands (at least four net positive charges present).

**3.3.4. Strong ICD (intensity of ICD band similar or stronger than the CD bands of DNA/RNA):** Usually of positive sign, strongly supports minor groove binding to DNA or major groove binding to ds-RNA (Figure 7, blue hue).

**Figure 8:**
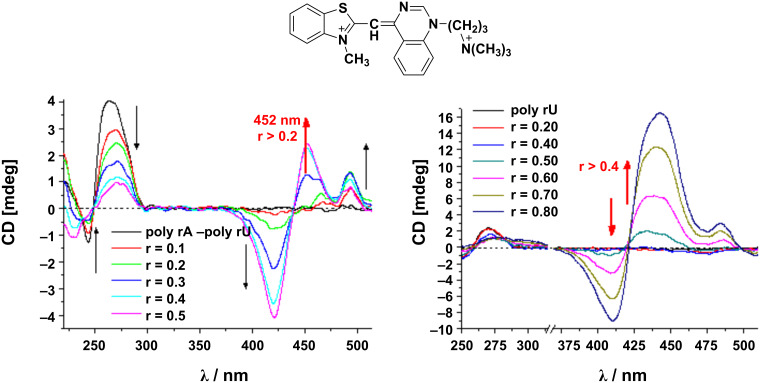
CD titration of poly rA-poly rU (left) and poly rU (right) (*c*(polynucleotide) = 2.0 × 10^−5^ mol dm^−3^) with ligand L at molar ratios r = [L]/[polynucleotide] (pH 7.0, sodium cacodylate buffer, *I* = 0.05 mol dm^−3^). Adapted from [30].

Exciton-coupled bisignate ICD bands: The appearance of exciton-coupled bisignate ICD bands (see chapter 2.1. and Figure 3, top) strongly support the aggregate binding along the polynucleotide, in which the ligand chromophores form an aggregate, with a well-defined supramolecular chirality. In this case, the ECD can still be said to be “induced” by the polynucleotide because it acts as a chiral template. An unambiguous interpretation of exciton-coupled bisignate ICD bands is not simple due to many different aggregation types which could take place upon DNA/RNA binding. For instance, at an excess of ligand over dominant DNA binding site (minor groove), surplus ligand molecules can form simple dimers within the DNA minor groove [28], which at even higher excesses over DNA can change to different, larger aggregates of H- or J-type [29]. In other cases the ligand can immediately form large helical arrays along the DNA or RNA as a dominant binding mode. For instance, a ligand upon binding to a polynucleotide can show ICD bands for several binding modes (Figure 8, left), whereby the first binding mode is dominant at r < 0.2, while the second binding mode (aggregation) is characterized by a new ICD band at 452 nm at r > 0.2. However, for another polynucleotide, the same ligand can give only one binding mode based on the uniform aggregation at excess of ligand over polynucleotide bases at r > 0.4 (Figure 8, right) [30].

**3.3.5. CD bands out of expected wavelength range:** In rare cases a ligand and DNA can form specific aggregates of very large sizes, which scatter light in the wavelength range where the components do not absorb light. In these conditions, a quite strong, well-defined and reproducible ECD band outside the expected range is observed. One example is the exceptionally strong CD spectrum of ψ-DNA [31–32], which is caused upon ligand addition (spermine) and by far exceeds the wavelength range at which the ligand and DNA absorb light (>300 nm). Such phenomenon is not related to standard chiroptical properties and will not be discussed here, but if a similar signal is obtained, it is advisable to refer to a set of methods dealing with DNA condensation (AFM, DLS).

### 4. Linear dichroism, LD

Circular and linear dichroism spectroscopy (ECD and LD) are often used as complementary tools for the investigation of DNA or RNA structure, as well as for DNA/RNA interactions with various ligands [2,6,9–10]. Figure 7 nicely summarizes the complementarity of the methods.

**Figure 9:**
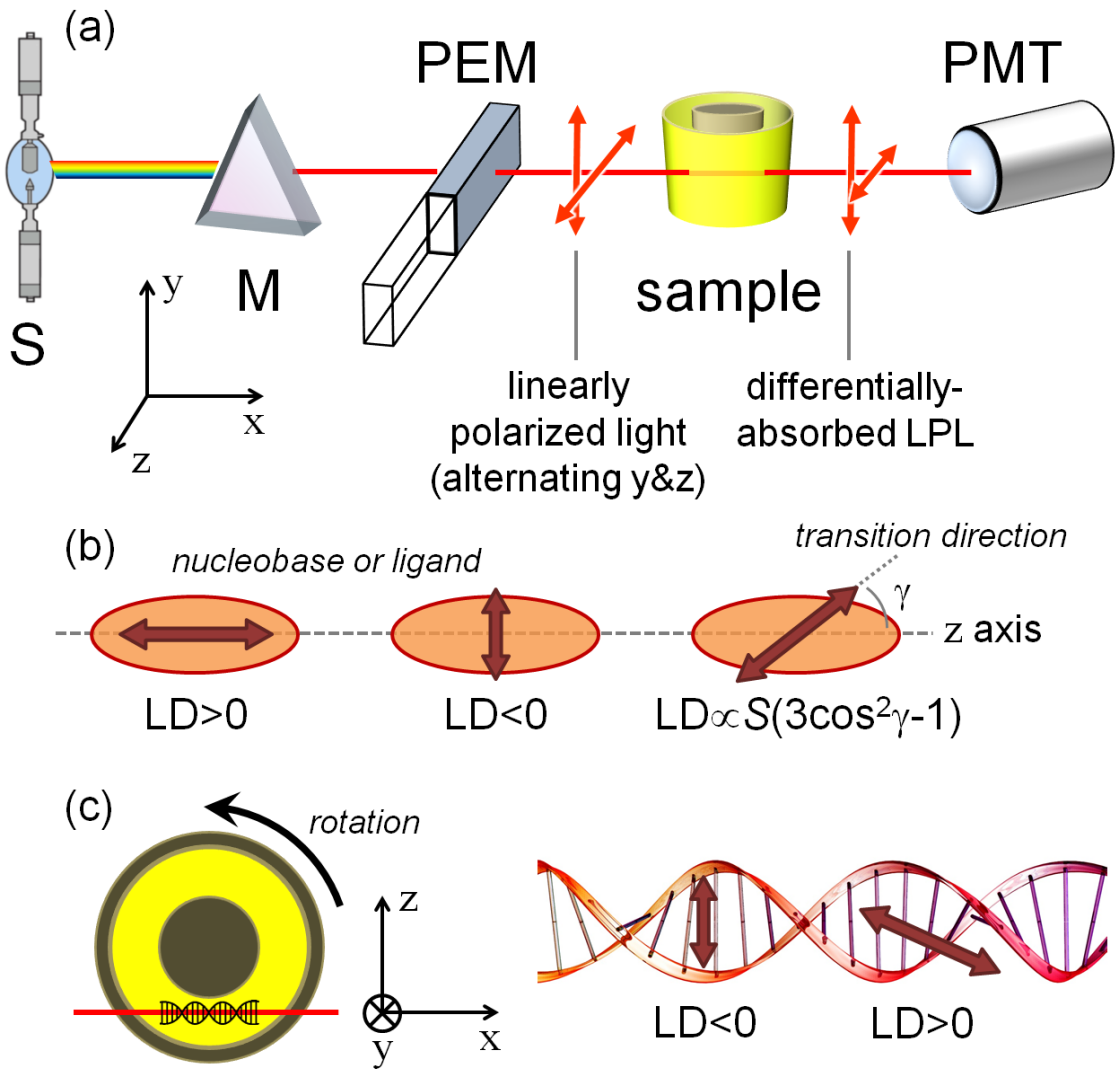
a) Schematic diagram of a flow LD instrument. b) Definition of the optical factor relating the direction of sample transition moment and the macroscopic sample orientation. The formula refers to a uniaxial sample; *S* is the orientation parameter, which quantifies the extent of effective sample orientation. c) Schematic illustration of a Couette flow LD experiment applied to a DNA/ligand complex.

As mentioned in the Introduction, the measurement of LD requires the sample to be oriented in a known way with respect to the polarization of radiation. Different orienting methods exist for different kinds of samples [2]. For macromolecules like polynucleotides which adopt a secondary structure with a preferred direction of elongation (that is, the polynucleotide helical axis), flow linear dichroism is the most suitable technique. In this case, DNA/RNA alignment is achieved by means of a special flow cell described below. Flow linear dichroism is defined as a difference in absorption of light polarized parallel and perpendicular to some reference axes taken by convention to be the flow direction along which the polynucleotide is at least partially aligned (Figure 9a). Flow LD is therefore a very useful method for the characterization of ds-DNA or ds-RNA conformation (base inclination), and of the flexibility and binding geometry of ds-DNA (RNA)/small molecule complexes [33–34].

**Figure 10:**
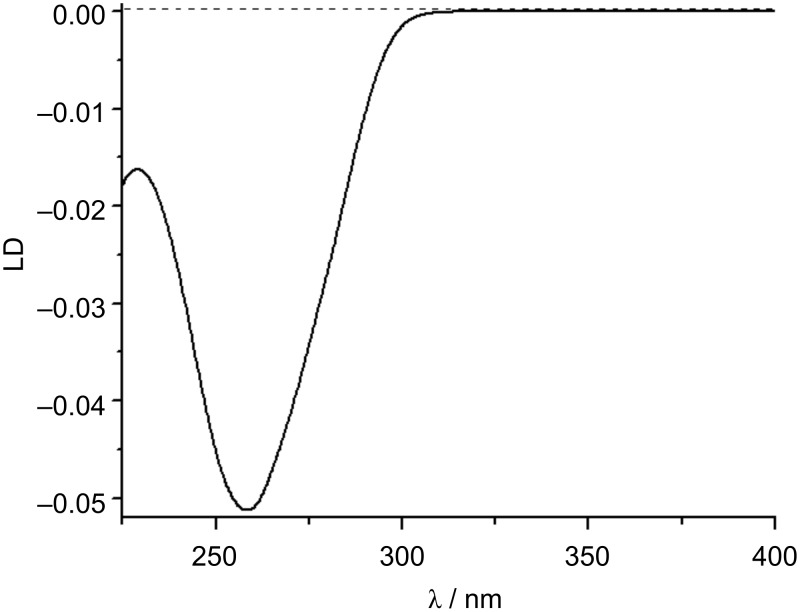
LD spectrum of *calf thymus* (*ct)* DNA (*c* = 2 × 10^−4^ M), pH 7, sodium cacodylate buffer, *I* = 0.05 M.

The aforementioned flow cell is a cylindrical rotating Couette cell, where the liquid is subjected to a constant gradient over the annular gap between the rotating (inner cylinder) and the fixed coaxial outer cylinder (Figure 9a and c). The speed of the rotation should be adjusted in such way as to cause the orientation of the molecule and not a turbulent flow and 5000 rpm is an indicative figure. A necessary prerequisite for a macromolecule such as DNA is a minimum length of at least 1000 base pairs to be successfully oriented [10]. LD probes the orientation of base transition moments relative to the DNA helical axis. Thus, standard B-DNA whose base pairs are perpendicular to the helical axis will have the same spectral shape as normal absorption spectra but with a negative sign in the 240–280 nm region of absorption of DNA (Figure 10). The negative sign stems from the definition of LD: LD = A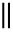 − A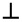, where A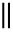 is the absorption for plane-polarized light parallel to the orientation axis, and A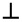 is the absorption for plane-polarized light perpendicular to the orientation axis. For a given ligand, according to induced LD (ILD), its orientation with respect to the DNA can be determined by flow LD as long as the direction of the transition dipole moment within the ligand is known or can be established, and the orientation parameter *S* (Figure 9b) of the DNA is also known.

#### 4.1. Practical information

A direct comparison of the experimental conditions and materials applicable in ECD and LD reveals that the LD method is more limited. Here are some reasons:

**Sample absorbance:** LD measures the absorbance only of the oriented parts of a sample (which can only be estimated), whereas most quantitative analyses require knowledge of the total absorbance.

**Polynucleotide length and sample concentration:** An efficient orientation along the flow requires a minimum length of the polynucleotide. It is estimated empirically that lengths over 1000 base pairs have a sufficiently high percentage of oriented DNA molecules to be measured at 100 µM concentrations (still 10 times higher than CD conditions). That precludes an LD application for any shorter polynucleotides, thus most of the synthetic DNA and RNA cannot be measured at *c* < 1 mM.

**Light scattering:** Non-flat baseline outside absorbing regions is a clear evidence of light scattering.

LD titrations are also collected as a function of the concentration of the compound. For that purpose, two types of flow cells are available: a cell with a total volume of 4 mL that allows adding stock solutions of the compound into the same cell, and 80 μL cuvettes for which every addition needs to be prepared separately.

For LD experiments, an additional spinning device is required. The baseline for LD experiments is measured on the solution in the non-spinning cuvette and should be recorded before each separate addition. All measurements should be done in the same cuvette which requires washing and careful drying between the measurements, often implying a long experimental time.

DNA should be at least 1000 base pairs long and therefore while preparing the DNA sample solution, sonicating is not recommended. The concentration of the DNA in the cuvette should be 100 µM. For the experiments described in the next section, it is relevant to understand that elongated cylinder-like polynucleotide fragments are spatially oriented along the liquid flow obtained by rotation of the solid quartz cylinder within the fixed outer quartz cylinder, separated by 0.5 mm.

**Instrument settings:** If the LD device is attached to the ECD spectrometer, for LD experiments the same settings can be adjusted as for the ECD experiments. Make sure to choose the LD option while choosing the channel mode in Data mode and adjust the bandwidth to 2 nm. The LD unit should be connected to the outer spinning motor.

#### 4.2. Practical/step by step

Steps, if using an LD unit with a total volume of 4 mL:

- Put 1 mL of a buffered solution of DNA (*c* = 200 µM) into the cell. To mix the solution in the cell, spin it by switching on the motor for a few seconds. Turn off the spinning and record the spectrum. Then, turn on the spinning up to 5000 rpm and record another spectrum. Subtract the non-spinning spectrum from the spinning spectrum to get the LD spectrum of DNA.
- Add an aliquot of the sample solution at a concentration which corresponds to the desired ratio r_[ligand]/[DNA]_ followed by the aliquot of DNA stock solution that will compensate for dilution. Mix the solution in the cell for a few seconds, turn off the spinning device and record the spectrum. After completion of the non-spinning spectrum, turn on the motor and record the spinning spectrum. Subtract the non-spinning spectrum from the spinning spectrum to get the LD spectrum at the first ratio r. Repeat this for all desired ratios r. While the spinning mode is on, check if there is any bubbling of the solution and monitor all the channels for scattering.

Steps, if using an LD cuvette with a total volume of 80 μL:

For this cuvette, it is advisable to prepare each solution containing free DNA and the desired set of ratios r_[ligand]/[DNA]_ separately in vials (with a total sample volume of 100 μL) and consequently transfer 80 μL of each sample into the cuvette, starting with free DNA, followed by samples from lowest to highest ratios r.

- Put 80 μL of a solution of DNA (*c* = 200 µM) into the cell. Mix the solution for a few seconds with spinning. Turn off the spinning motor and record the spectrum. Then, turn on the spinning up to 5000 rpm and record another spectrum. Subtract the non-spinning spectrum from the spinning spectrum to get the LD spectrum.
- After recording the first spectrum, the cuvette should be washed properly with redistilled water (3 times) and with ethanol (3 times, spectroscopic grade) followed by drying it using the air outlet.
- Into the dried cuvette, transfer 80 μL of a ligand/DNA mixture at a particular ratio r. Mix the solution, record the non-spinning spectrum and then the spinning spectrum. Repeat this for all samples.

#### 4.3. Interpretation of the results

If a targeted biomacromolecule is spatially well-oriented in the sample, a small molecule which binds uniformly to identical, mutually independent binding sites of the biomacromolecules, will acquire an LD signal with an intensity proportional to the quantity of bound small molecules [33–34]. However, the intensity of the LD signal depends on several other factors such as: a) the orientation parameter of the DNA (*S* in Figure 9); b) the orientation of the bound molecule with respect to the macromolecule (Figure 7); c) the local direction of the transition dipole moment allied with the observed absorption band (Figure 7). Provided that at least factor *S* is known, one may in principle use LD measurements to estimate the angle by which a transition of a bound molecule is oriented relative to the axis of the DNA helix.

**4.3.1. Changes in DNA absorbing region (<300 nm):** The LD spectrum of ds-DNA/RNA can change upon binding of small molecules due to structural changes of the double helix. For instance, if upon small molecule binding the DNA/RNA double helix is shortened or kinked, it will orient less effectively and the LD signal will be reduced and/or changed in its shape [33–34]. Alternatively, a marked increase of the negative LD amplitude at 260 nm with the addition of a small molecule could imply that the DNA/RNA becomes better-oriented in flow, due to a stiffening of the ds-DNA/RNA structure [33–34].

**4.3.2. Induced LD (>300 nm):** When the compound is added to the DNA, and if the compound’s absorbing region shows LD signals, the compound is binding in one or more specific orientations (rather than randomly along the backbone) [6,10,35]. The sign of the induced LD then can give information on the geometrical orientation of a bound compound to the DNA (Figure 7):

1. Negative ILD: Negative ILD in the compound’s absorbing region indicates that the transition moment of the compound, allied with the observed absorption band, is perpendicular to the DNA axis (90°), which is consistent with intercalation [2,6].
2. Positive ILD: When a positive ILD is observed for the polarized band of the compound, this implies that at least the chromophoric portions that are associated with the observed transitions of the compound are not intercalated (inserted between the base pairs, thus being perpendicular to the DNA chiral axis). Instead, the direction of the chromophore transition moment (e.g., the chromophore long axis indicated by the blue double-arrow in Figure 7) is most probably oriented at about 45° to the DNA chiral axis, thus matching the minor groove of the DNA [6,10]. For a planar molecule, this implies non-intercalative binding, whereas, for many potentially nonplanar or flexible molecules, a partial insertion between base pairs cannot be excluded on this basis.
3. No or very small ILD: The compound is non-specifically oriented, either due to a mixed binding mode or externally bound to the phosphate backbone.

### 5. Vibrational circular dichroism, VCD

VCD is analogous to ECD in the IR region of the electromagnetic spectrum, where molecular vibrational transitions occur (between 4000 and 750 cm^−1^) [3,36]. Recently, VCD has developed as a reliable spectroscopic method for the determination of the absolute configuration and conformational distribution of chiral molecules in solution [1,4,36]. The main drawback of VCD is the inherently small signal intensity, which is around 100-fold less intense than for ECD. Despite recent technical improvements, for accurate results highly concentrated samples are still needed. The insufficient solubility of DNA or RNA samples, as well as aggregation properties often hamper an application of VCD. Another severe problem is the need for an IR-transparent solvent, which hinders measurements in purely aqueous solutions, if not done in D_2_O. The main advantage of VCD over ECD is that it does not need the presence of a conjugated aromatic chromophore, making VCD spectra much richer in bands than a typical ECD spectrum (just like IR spectra vs UV–vis spectra) [1,4,36].

Nowadays, density functional theory (DFT) allows a calculation of the VCD spectrum of a chiral molecule, which then can be compared with the measured VCD spectrum and by this the absolute configuration (AC) of a chiral molecule can be determined [37]. Furthermore, an oligonucleotide VCD spectrum calculation was described recently [38].

#### 5.1. DNA VCD

In VCD spectroscopy, two major spectral regions can be monitored to probe the structures of nucleic acids. In the region between 1700–1600 cm^−1^, the stretching modes of C=O, C=N and C=C of the nucleic acid bases occur. The vibrational bands appearing above ≈1650 cm^−1^ are associated with the C=O (due to different number of these groups found in different bases, nucleic acids with different base compositions will provide different VCD spectra) and those appearing below ≈1650 cm^−1^ are associated with the C=C stretching modes (with some contribution from C=N stretching as well) [39–40]. The VCD in this region is highly sensitive to the secondary structure of DNA/RNA and may be employed to assess secondary structures and to monitor their changes upon various stimuli [41]. Similar to the ECD case, such a correlation is often made on an empirical basis [39–40] but may be substantiated by theoretical approaches [38].

The other spectral region interesting for nucleic acids is 1250–1000 cm^−1^, where the stretching modes of the phosphate group are found plus some vibrational modes of the sugar moieties. The VCD appearing in this region is less dependent on the base composition [42].

#### 5.2. Practical information

**Choice of solvent and sample cell:** Although the analysis of biomolecule interactions is best performed in aqueous media, water is not the most suitable solvent for VCD due to its strong band (“scissoring” mode) found at 1650 cm^−1^, overlapping with the C=O stretching from nucleic acids [35]. Therefore, highly pure deuterated water (D_2_O) is used for VCD DNA measurements. The frequency range that is not accessible in D_2_O is 1150–1450 cm^−1^ [39]*.* In that case, ordinary NaCl and KBr sample cells cannot be used due to their solubility in water. Instead, CaF_2_ or BaF_2_ cells are used in VCD measurements. CaF_2_ allows measurements down to 1000 cm^−1^ while BaF_2_ is used for measurements for which a wider spectral range down to ≈750 cm^−1^ is needed. Special care must be taken regarding storing and handling of the cell because of the slight hygroscopicity of BaF_2_. Standard cells for VCD comprise two CaF_2_ or BaF_2_ windows separated by a Teflon spacer and sealed, with path lengths around 50–100 μm [36]. They have small volumes and must be filled carefully using syringes, making the addition of aliquots not practical for VCD.

**Sample preparation and instrument adjustments:** Sample concentration at different ratios r in pure D_2_O should be prepared possibly under anhydrous conditions and inserted into the cells. IR absorbance adjusted to around 0.4 is optimal for VCD measurements [36]. This corresponds to a high sample concentration, which is unfavorable for the measurement of rare biomolecules.

Contrary to ECD, VCD instruments use Fourier transform (FT) collection of spectra. The standard resolution is 4 cm^−1^ [37]. Because of the small signal-to-noise ratio, several thousand spectra need to be collected and averaged (at least 2000–4000). This requires long measurement times, usually 1 h or longer. VCD spectra must be baseline corrected by subtracting the solvent spectra from those of the samples. However, a baseline drift is expected to occur over long acquisition times.

**VCD applications to study DNA or RNA/ligand binding:** Because of several reasons which should be clear from the paragraphs above, VCD is much less employed than ECD in the study of adducts between DNA/RNA and ligands. Another reason is that, due to the frequently very complex pattern of bands seen in VCD spectra, a clear-cut division into regions where only the polynucleotide or only the ligand contribute is impossible, contrary to what happens for ECD. Therefore, we will briefly mention here a few illustrative applications. An extensive review has been published in 2009 by Urbanová [11].

By far the best investigated ligands by VCD are porphyrins. Studies with both natural and synthetic DNA allowed the authors to establish the dominant binding modes, helix distortion, and base-pair stabilization [43–44]. In these studies, the diagnostic signals were those associated with C=O stretching and interestingly enough, VCD was always used in combination with ECD. We wish to mention that the interaction of nucleic acids and metal porphyrins can also be studied by magnetic circular dichroism (MCD), a chiroptical spectroscopy based on the differential absorption of L-CP and R-CP light in the presence of a strong magnetic field oriented parallel to the direction of light [45]. Another well-studied ligand is the anticancer drug daunomycin, proving its preferred intercalation site [46]. Finally, we mention a study on cisplatin bound to a model DNA octamer, whose VCD spectra were simulated by density functional theory (DFT) calculations [47].

### 6. Emission-based polarized spectroscopies: Fluorescence detected circular dichroism (FDCD) and circularly polarized luminescence (CPL)

#### 6.1. General

Emission-based spectroscopy methods such as fluorescence-detected circular dichroism (FDCD) and circularly polarized luminescence (CPL) combine the advantages of both chiroptical and fluorescence techniques, therefore, being sensitive to molecules which are both chiral and fluorescent [3,48].

**Figure 11:**
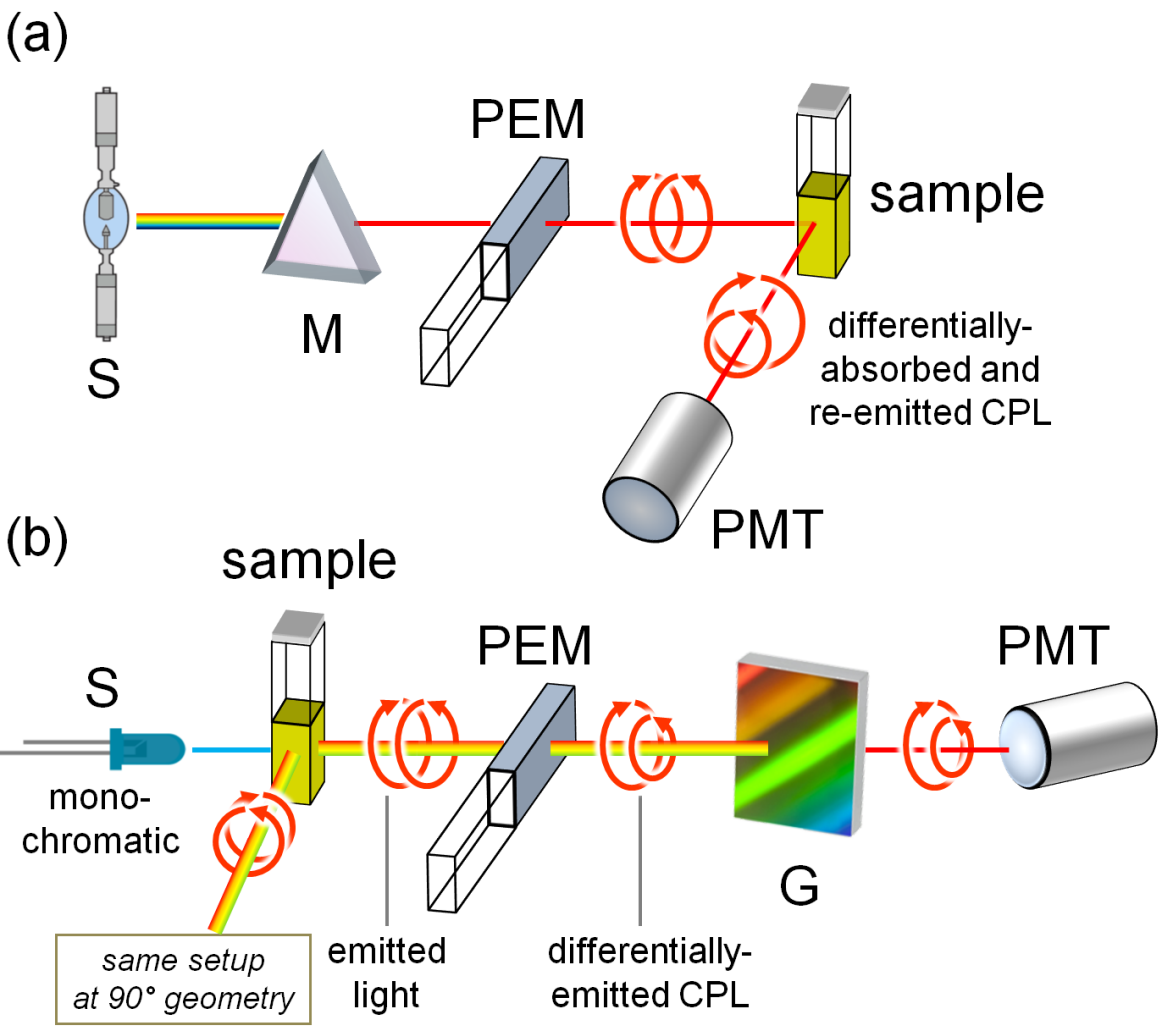
Schematic illustration of an FDCD a) and a CPL b) instrument. Legend: S, source; M, monochromator (excitation wavelength selector); G, diffraction grating (emission wavelength selector); PEM, photoelastic modulator; PMT, photomultiplier (detector). In CPL, both 0° and 90° setups are possible; in FDCD, the 90° setup is used.

The FDCD method is based on the collection of the differential fluorescence emission of a sample excited alternatively by left and right circularly polarized light (Figure 11a). Combining the conformational sensitivity and chiral specificity of ECD with the detection sensitivity and specificity of fluorescence, FDCD is far more specific and sensitive than standard transmission ECD [3,49]. CPL is the differential emission of left and right circularly polarized radiation from a chiral fluorescent sample irradiated with non-polarized light (Figure 11b). FDCD and CPL represent the chiroptical counterparts of fluorescence spectroscopy recorded in the excitation and emission mode, respectively. Therefore, while FDCD senses the geometry and properties of the ground state, CPL senses those of the lowest excited state [49].

FDCD was used for measuring the mixtures of a fluorophore and one or more chromophoric but non-fluorophoric species, whereby it was possible to determine the contribution of only the fluorescent probe in the system [49], like for instance, a fluorescent ligand associated with a biopolymer or a fluorophore inserted into non-fluorescent chiral biomolecules (DNA, RNA, proteins) [3]. In such cases, FDCD monitors specifically the fluorescent ligand, provided that no energy transfer occurs. Additional advantages are that it can be used on samples that are optically dense and/or highly scattering [50]. The latest applications of FDCD to detect ligand/nucleic acid interaction date back to the ’80 [51], mostly because of the technical difficulties explained below. On the contrary, CPL is still in its infancy regarding the same context. The first paper describing the interaction of *ct*-DNA with two fluorescent ligands has recently appeared [12]. CPL may complement ECD by providing specific information on the emission behavior of a fluorescent achiral ligand bound to DNA.

#### 6.2. Some practical advice and guidelines for FDCD and CPL experiments

FDCD experiments can be done in a fluorescence cuvette with a 1 cm path length in an ECD instrument with a photomultiplier placed at 90° to the excitation beam to collect ECD and fluorescence from the sample simultaneously. The emission signal should be filtered by using a long-pass filter, or, alternatively, by an emission monochromator. The most important drawback of FDCD measurements is the presence of so-called polarization artifacts, related to the presence of fluorescence polarization (photoselection) [52]. These are expected to be especially important for nucleic acids and their adducts because of the size of the system. An ellipsoidal mirror surrounding the cell can be used to improve the collection of fluorescence emission and to avoid artefacts [50].

CPL is also subjected to various kinds of artifacts, which need to be properly considered [12]. It must be stressed that the first commercial CPL instrument has only recently appeared on the market, sold by Jasco, Inc., but several home-made instruments are active.

### 7. Analysis of the obtained results

It is essential to remember that the here presented methods are often not sufficient by themselves for the accurate characterization of a ligand/polynucleotide complex, but usually are part of a broader set of methods (including fluorimetric and/or UV–vis titrations, thermal denaturation experiments, isothermal titration calorimetry (ITC) experiments, gel electrophoresis, etc.) [6]. Thus, the interpretation of the results should take into account all data.

#### 7.1. Binding constant and binding ratio n_[ligand]/[polynucleotide]_ determination

Traditionally, the affinity of the ligand to DNA or RNA is calculated from the Scatchard equation (McGhee, von Hippel formalism) [24], advisably by non-linear fitting of the experimental data (to avoid numerous problems, like large data-weighing errors, associated with linear Scatchard transformations) [53], and presuming that a single dominant binding site occurs for each ligand. An excellent protocol on how to organize CD experiments and data processing is given in reference [9]. One of the intriguing new approaches is GlobalFit processing [54–55], whereby the data from all titration experiments (e.g., CD, fluorescence, ITC, etc.), done at approximately similar conditions (concentration range, buffer), are processed simultaneously. The advantage is not only related to the use of a broad set of mutually independent methods, which can hardly have the same artifacts, but also the different sensitivity of each method for a particular complex formation response. For instance, a weak ICD band showing a non-linear change in titration experiments could be an error of the method, but if the non-linear fitting in GlobalFit procedure agrees with ITC or fluorimetric titration, then the ICD band can safely be attributed to a bound ligand.

However, if the ligand binds to DNA/RNA by multiple different binding modes within the same titration experiment (which is also detectable by other methods such as fluorescence and ITC), then Scatchard-based non-linear fitting cannot provide binding constants for all binding modes, but only for the prevalent binding at high excess of DNA/RNA over ligand, at which each ligand is bound independently. One of the possible approaches was demonstrated on the model (distamycin A and netropsin), showing how ECD data can be used in deconvolution of complex systems [56]. Also, singular value decomposition (SVD) analysis could help, since it allows a model-free determination of the number of linearly independent components in a given matrix of data [57]. Furthermore, several commercially available programs (e.g., HyperQuad [58] or Specfit [59]) offer versatile approaches to multicomponent spectra analysis, whereby the introduction of at least some known parameters (binding constants for dominant binding sites derived from GlobalFit or Scatchard calculations) allows the deconvolution of ECD or LD data to give ICD (ILD) bands for each type of complex. Unfortunately, the intensity and the sign of ICD are hardly predictable, and in such cases, the computational approach given below can help.

#### 7.2. Computational approach – a focus on ECD spectra

The use of computational approaches in the study of polynucleotides and their adducts with ligands may appear a formidable task if one looks at the complexity of the system. In fact, the calculation approach must rely on relatively small models and ad hoc calculation strategies. In the past, the first theoretical descriptions of ECD spectra of polynucleotide/ligand complexes were based on the coupled-oscillator model [60–62] or on the so-called matrix-method approach (which also includes magnetic-allowed n–π* transitions) [63–64]. These theoretical works were essential to substantiate the relation between ECD spectra and the mode of binding (see chapter 2, interpretation of results), first established empirically. The coupled-oscillator and matrix methods are hybrid in the sense that they require the knowledge of the chromophore transitions either from the literature or from quantum mechanical (QM) methods. The transitions are then described by a set of parameters (position of the point dipoles, dipole orientation, dipolar strength) and are allowed to “interact” through classical electrostatic equations, for example, the Coulombic dipole/dipole potential. Although the basic fragmentation or many-body approximation is retained in some modern calculations methods for DNA/RNA [65–67], these latter rely explicitly on QM computations such as time-dependent density functional theory (TDDFT) [68]. Alternatively, more time-efficient QM schemes like the so-called sTDDFT (simplified TDDFT) and sTDA-xTB (simplified Tamm–Dancoff approximation with extended tight binding) can be directly employed on relatively large molecular systems like DNA [69].

The main aim of QM ECD calculations of complexes between DNA/RNA and ligands is to simulate an ECD spectrum to be compared with the experimental one. Of course, the absolute configuration is not an issue here, while the overall geometry is. Ultimately, then, the aim of such calculations is to substantiate the occurrence of a certain binding mode which has been (empirically) assessed by ECD and/or LD, or other spectroscopic techniques [70]. A typical ECD calculation will require three major steps: a) the selection of an appropriate model, b) the generation of an input structure, and c) the actual ECD calculation.

**Figure 12:**
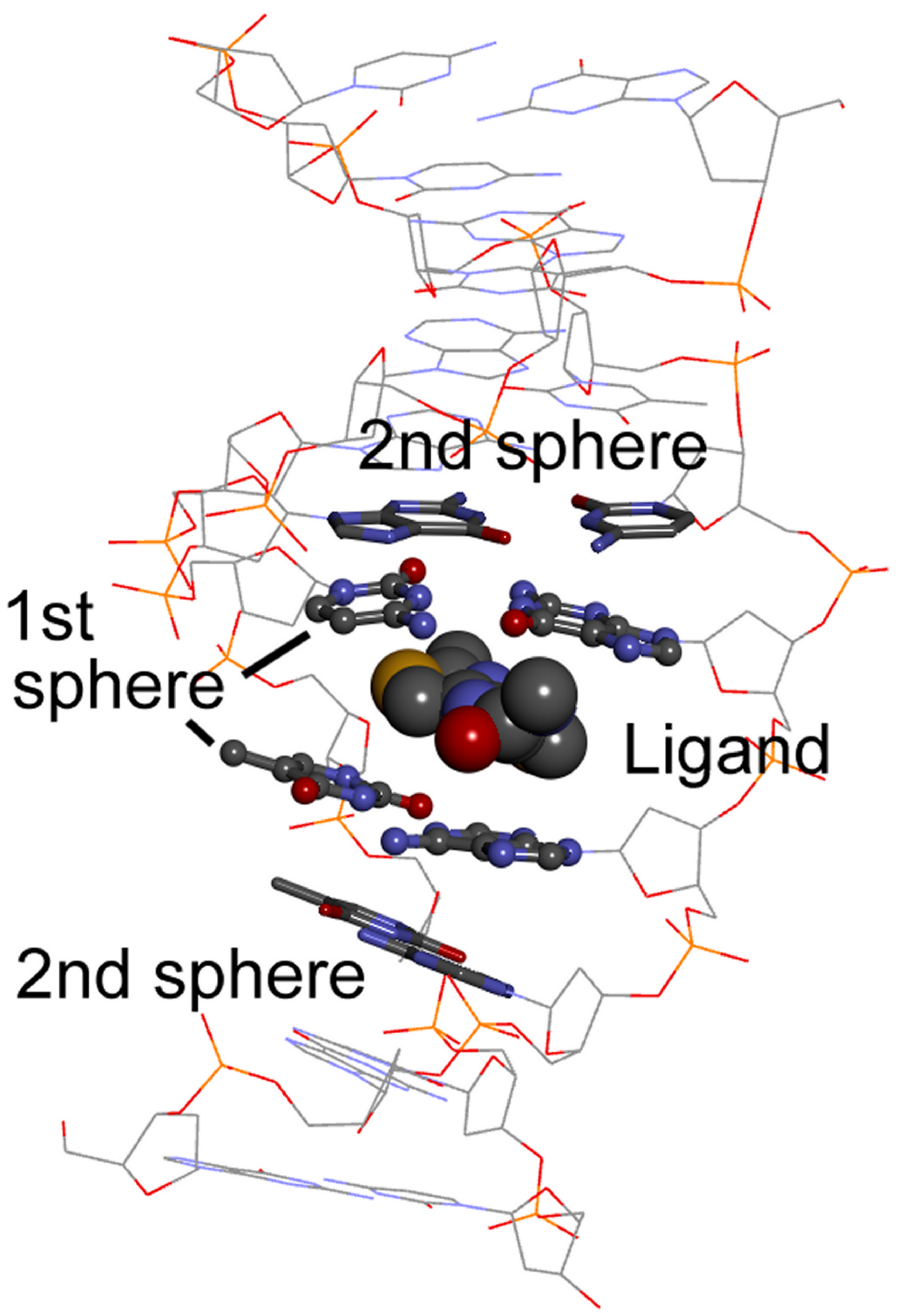
Minimal model to be considered to simulate the ICD spectrum of an intercalated ligand. ICD will be dominated by the intrinsic chirality of the ligand plus the nondegenerate exciton coupling with the most immediate nucleobase pairs (1st sphere). The following nucleobase pairs (2nd sphere) may possibly be neglected in the ECD calculations, but not in the input geometry generation.

**a) Selection of the model:** It is obvious that a full QM calculation of a “real” system made of a large portion of DNA/RNA helices, counterions, solvating and surrounding water molecules, plus one or more bound ligand molecules, is not affordable even with state-of-the-art computers. Therefore, one must select an appropriate system to be handled at least with the simplified TDDFT or TDA approaches mentioned above, or even with standard TDDFT. This necessarily requires the DNA/RNA to be replaced with a short oligomer, which in turn means that one must focus only on the ICD in the region >300 nm (see chapter 3). ECD, and especially exciton coupling, is dominated by first-neighbor interactions [21], therefore one needs to consider the nucleobases closest to the ligand. For example, for an intercalated ligand one may consider the two base pairs involved in the intercalation and their immediate neighbors, thus focusing on a basepair tetramer (Figure 12). For groove binders, the number of base pairs will be necessarily higher. Counterions are often neglected while water molecules may be important especially if the ligand has the option to act as hydrogen-bond acceptor or donor.

**Figure 13:**
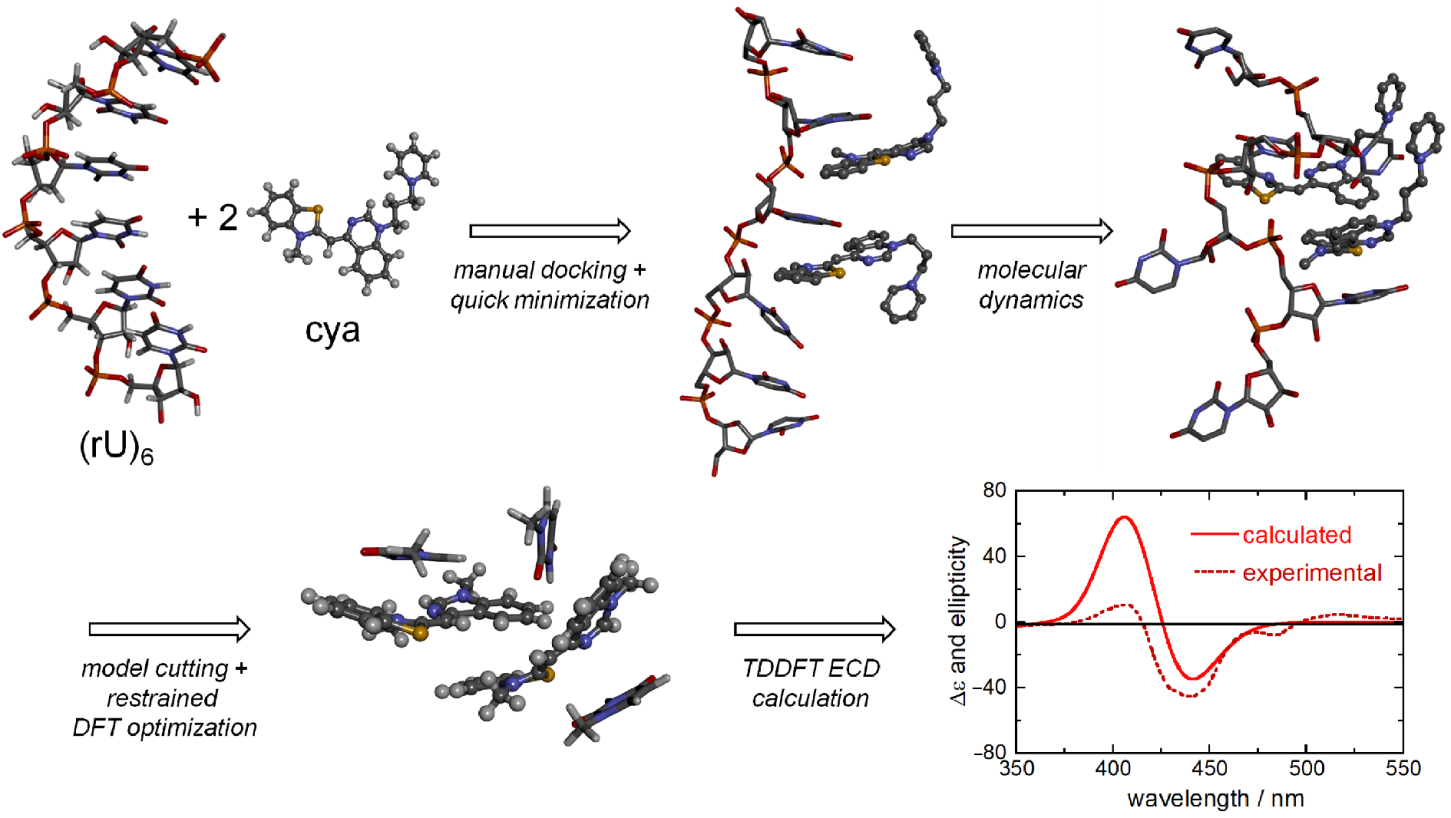
Schematic representation of the main steps for calculating the ICD spectrum of a ligand bound to a single-stranded RNA. In this case, a double binding of cyanine ligands (cya) was expected to occur in two consecutive binding sites. In the final model for ECD calculations, two “cya” molecules and the three closest nucleobases found after MD were considered. The ICD is exciton-like and is dominated by the exciton coupling between the cyanine ligands. Adapted from [30].

**b) Input structure:** This is probably the most intriguing step of the process and can be divided into three sub-steps. In the first sub-step, one must obtain a molecular model of the DNA/RNA in the conformation evidenced by previous experiments. The most obvious source thereof would be PDB database. Then, a molecular model of the ligand molecule can be generated by using a molecular-modeling software. At this point, one must model the binding. A naive approach which can be tried as first choice would be to manually dock the ligand to the desired site (intercalation, minor or major groove) and then to relax its structure and that of the closest base pairs by molecular mechanics calculations with a good force field [71], by keeping the remaining structure fixed (first step in Figure 13). Most often, however, this approach is not accurate enough, and one must resort to the more typical methods used for modeling of polynucleotides. These include docking simulations and/or molecular dynamics (MD) simulations, possibly within a solvent cavity (second step in Figure 13). The description of these methods is outside the scope of the present tutorial and specialized books should be consulted [72–73].

In the second sub-step, one must simplify the structure obtained in the previous step (which would be by far too large for any QM approach) by cutting all unnecessary parts which are not expected to make a sizable contribution to the ligand ICD (see point a) above), neither to change much the structure of the ligand and of the surrounding nucleobases. Looking at Figure 12, one will preserve the ligand, the “first sphere” of nucleobases closest to the ligand, and the “second sphere” of nucleobases which are necessary to keep the ligand plus the first sphere in their position. Of course, two or more ligand molecules will be needed if multiple binding occurs (Figure 13) and relevant water molecules, hydrogen-bonded to the ligand, must be included.

In the third sub-step, the geometry of ligand(s) and the first sphere must be optimized with an accurate QM level, for example, DFT with a good functional such as M06-2X or ωB97X-D (third step in Figure 13). The second sphere can be kept frozen, or at least a part of it can be treated at a lower level of theory using the ONIOM approach. This optimization step is necessary to obtain a more accurate geometry of the chromophores which will be used for the ECD calculations, however, it shouldn’t alter too much the overall geometry of the restricted model with respect to the whole system obtained after the first sub-step.

**c) ECD calculations:** Finally, ECD calculations will be run (last step in Figure 13) with one of the methods discussed above, in order of decreasing machine time usage: full TDDFT, sTDDFT, sTDA-xTB, TDDFT-based fragmentation approaches. For details, we refer the reader to the literature cited above. In the actual calculations, one may entirely neglect the second sphere or include it with some embedding approach like ONIOM. The QM system should be as small as possible, including the ligand(s) plus the closest nucleobases (bottom structure in Figure 13) [30], but even only a small ligand aggregate after the templating effect of DNA has been considered to build its geometry [74].

The calculated ECD spectrum resulting from the above sequence will be then compared with the experimental one, focusing only on the portion of the spectrum allied with the ligand transitions. It is important to compare not only the sign but the overall shape including the intensity. ECD is extremely sensitive to geometry, especially to the reciprocal arrangement of multiple chromophores. Therefore, in case of a good match, this will be a strong indication that the tested binding mode is the correct one. Otherwise, one can try if a different binding mode leads to a better agreement, with a trial-and-error tactic. On the other hand, it must be stressed that the described approach is based on several approximations, especially in the generation of the input structure. This means that a perfect agreement between the experimental and the calculated ECD may not necessarily be obtained even for the correct binding mode.

## Conclusion

The family of chiroptical spectroscopy methods offers a toolbox of techniques which are very useful to characterize not only biomacromolecules such as nucleic acids, but also their interactions with small molecules. These techniques are based on the differential absorption or emission of circularly polarized light, thus they suffer from intrinsic lower sensitivity than their counterparts not based on polarized radiation. At the same time, however, they feature greatly enhanced structural sensitivity and selectivity toward chiral species. Therefore, they lend themselves as very practical tools to detect adducts between nucleic acids and small molecules, follow their evolution upon external stimuli, quantify their thermodynamics and kinetics, and provide information about the mode and geometry of binding. With the further development of computational methods, moreover, it is expected that spectra-to-structure relationships will be analyzed with greater accuracy and offer a detailed snapshot of the biomacromolecule/drug interaction at the molecular level.
